# Psychometric properties of the German Hogg Eco-Anxiety scale and its associations with psychological distress, self-efficacy and social support

**DOI:** 10.1186/s12889-025-22849-3

**Published:** 2025-05-02

**Authors:** Lara Denise Henschel, Gabriele Helga Franke, Melanie Jagla-Franke

**Affiliations:** 1 Hochschule Magdeburg-Stendal, 39576 Stendal, Germany; 2 Hochschule Neubrandenburg, 17033 Neubrandenburg, Germany

**Keywords:** Eco-anxiety, Climate anxiety, Mental health, Psychological distress, Psychometrics

## Abstract

**Background:**

The present study aimed to investigate the psychometric properties of the German Hogg Eco-Anxiety Scale (HEAS) as a reliable and valid instrument to measure eco-anxiety, and to explore its associations with sociodemographic and psychological variables.

**Methods:**

322 German speaking participants (67.4% female; M = 36.64 [SD = 14.77] years old) were recruited via the internet and social media. Confirmatory factor analyses, reliability and correlational analyses, independent sample t-tests, and a multiple regression analysis were conducted.

**Results:**

Both in confirmatory factor analyses tested models were acceptable with an even better model fit of the four-factorial structure (CFI = 0.96, TLI = 0.94, RMSEA = 0.08, SRMR = 0.04) than of the second-order model (CFI = 0.94, TLI = 0.93, RMSEA = 0.08, SRMR = 0.06). The HEAS total scale (α = 0.91, ω = 0.91) and the HEAS subscales Affective Symptoms (α = 0.87, ω = 0.86), Rumination (α = 0.84, ω = 0.84), Behavioral Symptoms (α = 0.79, ω = 0.79) and Personal Impact Anxiety (α = 0.90, ω = 0.90) had good to excellent internal consistency coefficients. Correlational analyses showed significant associations between the HEAS total scale and subscales and measures of climate anxiety, psychological distress, partially self-efficacy and social support as well as some sociodemographic variables. Some significant sociodemographic differences were found for the HEAS total scale and subscales regarding gender and parental status but not age groups. Our multiple regression analysis resulted in psychological distress as the only significant predictor of eco-anxiety.

**Conclusion:**

The German HEAS is a reliable and valid instrument to assess anxiety about ecological problems.

## Background

Within the existing literature, the diverse terms used to describe the negative affects resulting from the perception and experience of ecological crises– including climate anxiety and eco-anxiety– are often used synonymously but may differ conceptually [1]. Climate anxiety is an emotional reaction in view of the real threat to individual and collective well-being posed by climate change [2]. Eco-anxiety is ascribed a broader meaning compared to climate anxiety, which is not limited to one environmental issue such as climate change [1]. In the following, however, both terms should be understood as synonyms, so that “eco-anxiety” is used. Eco-anxiety can have a maladaptive effect and not lead to the desired pro-environmental behavior to mitigate climate change [3]. The ability to deal with eco-anxiety in an adaptive way so that it motivates action may be related to the degree of impairment [4]. In this sense, it is speculated that there is not a linear but an inverted U-shaped relationship between eco-anxiety and pro-environmental behavior, such that moderate levels of anxiety are associated with optimal levels of pro-environmental behavior, while both low and high levels of anxiety are associated with less engagement in pro-environmental behavior [5, 6]. To better understand eco-anxiety, so that it can be used as a motivator of the desired behavior, this paper aims to contribute to the current state of research on the phenomenon of eco-anxiety by examining the psychometric properties of the Hogg Eco-Anxiety Scale [7] as a frequently used instrument for measuring eco-anxiety as well as the relationships between the construct and sociodemographic and psychological variables in a German-speaking sample.

### The Hogg Eco-Anxiety Scale

The Hogg Eco-Anxiety Scale (HEAS) [7], originally developed in New Zealand, quantifies affective, ruminative, and behavioral indicators of eco-anxiety as well as anxiety about one’s own personal negative impact on the planet, by using 13 items. The HEAS has been so far translated and validated in Arabic (ω = 0.65-0.82) [8], French [9], German (α = 0.71-0.86) [10], Italian (α = 0.78-0.86) [11], Polish (α = 0.79-0.85) [12], Portuguese (α = 0.85-0.92) [13], Spanish (α = 0.71-0.81) [14] and Turkish (α = 0.83-0.91) [15]. The four-factorial structure of the HEAS, consisting of the factors Affective Symptoms, Rumination, Behavioral Symptoms and Personal Impact Anxiety found in the original study by Hogg and colleagues (α = 0.86-0.92) [7] has been replicated across various studies [8–18]. In the French two-wave longitudinal study of Pavani, Nicolas, and Bonetto [19], their CFA suggested that the one-factor-solution of the HEAS fit the data well (see Table 1), so that they computed a single indicator of eco-anxiety by calculating the mean value across all 13 items, which was internally consistent (α_t1_ = 0.91, α_t2_ = 0.94). In contrast, testing a one-factorial model in the studies of Çimşir and colleagues (α = 0.74-0.87) [16], Larionow and colleagues [12] and Rodríguez Quiroga and colleagues [14] resulted in a poor model fit (see Table 1). Also, the second-order model with the four HEAS subscales as first order factors tested by Larionow and colleagues [12] was inferior to the four-factor model (see Table 1). The HEAS has previously shown measurement invariance between genders in Portuguese [13] and Turkish adults [16], between genders and age groups in Australian adults (α = 0.78-0.88) [17], and at gender, age, and country levels in Spanish and Argentinian samples [14].

**Table 1:** CFA fit indices of tested one-factorial and second-order models of HEAS

		CFI	TLI	RMSEA (90% CI)	SRMR
Pavani et al. [19]	One-factorial	0.93	0.91	0.08	0.08
Cimsir et al. [16]	One-factorial	0.66	0.59	0.16 (0.15-0.17)	-
Rodriguez Quiroga et al. [14]	One-factorial Argentina/Spain	0.63	0.55	0.17 (0.17-0.18)	0.13
	One-factorial Argentina + Spain	0.66	0.59	0.15 (0.15-0.16)	0.11
Larionow et al. [12]	One-factorial	0.56	0.48	0.21 (0.19-0.22)	0.15
	Second-order	0.92	0.90	0.09 (0.08-0.10)	0.11

CFI: Comparative Fit Index. TLI: Tucker-Lewis Index. RMSEA: Root mean standard error of approximation. CI: Confidence Interval. SRMR: Standardised root mean squared residual

### The German version of the Hogg Eco-Anxiety Scale

As mentioned above, there is already a translated and validated German version of the HEAS by Heinzel and colleagues [10], who tested the scale with 486 students and university staff in Germany (α = 0.71-0.86). In their study, they assessed the psychometric properties of the HEAS and investigated whether their data supported the original four-factorial structure of the scale. The authors performed a Bayesian CFA, which showed a good model fit for the four-factorial solution with minor cross-loadings of items from the factors Rumination, Behavioral Symptoms and Personal Impact Anxiety on the Affective Symptoms factor. This four-factorial model also showed a better fit to their data than the alternative three-, two- and one-factorial solutions they tested. For comparability, Heinzel and colleagues [10] also tested the four-factorial model in a conventional CFA, which resulted in a good model fit (CFI = 0.98, TLI = 0.98, RMSEA = 0.06 [90% CI, 0.04-0.07], SRMR = 0.02). Correlational analyses results indicated the distinction of eco-anxiety dimensions and general depression, anxiety, and stress. The result of their multiple linear regression showed that only the subscales Affective Symptoms and Behavioral Symptoms were related to general anxiety.

### Sociodemographic influences on eco-anxiety

Studies have shown that women report significantly higher values on the scales Affective Symptoms and Personal Impact Anxiety than men [11, 14, 16, 18]. In addition, women in the studies of Rodríguez Quiroga and colleagues [14] and Türkarslan and colleagues [18] showed significantly higher levels of rumination than men, whereas this was observed the other way around in the study of Çimşir and colleagues [16]. Ali and colleagues [8] only found significant differences on the subscale Affective Symptoms, whereas Larionow and colleagues [12] only found significant differences on the subscale Personal Impact Anxiety, with women reporting higher values than men. However, in the Portuguese study no gender differences emerged [13].

Rodríguez Quiroga and colleagues [14] found significant small negative associations between age and the HEAS subscales Affective Symptoms, Behavioral Symptoms and Personal Impact Anxiety. Ali and colleagues [8] also found significant small negative associations between age and the HEAS subscales Behavioral Symptoms and Personal Impact Anxiety. Larionow and colleagues [12] reported significant small negative associations between age and the HEAS subscales Affective Symptoms, Rumination, Behavioral Symptoms and Personal Impact Anxiety. The Australian study by Hogg and colleagues [17] found that age was weakly associated with experiencing more rumination and less anxiety about one’s personal negative impact on the planet but did not correlate with the experience of affective and behavioral symptoms. In the study by Rocchi and colleagues [11], people aged 30 and over scored significantly higher on the Affective Symptoms and Personal Impact Anxiety subscales than people under 30 years of age. In the study by Rodríguez Quiroga and colleagues [14], younger people also showed significantly higher scores on the subscales Affective Symptoms, Personal Impact Anxiety, and, additionally, Behavioral Symptoms. On the other hand, Türkarslan and colleagues [18] found no age group differences between people aged 25 or younger and people over 25 years of age. These results suggest that the links between eco-anxiety and age are mixed and need to be further explored.

Jalin and colleagues [20] found that being female, having a high level of education and not having children predisposed to a higher level of eco-anxiety in the Eco-Anxiety Measurement Scale [21]. In the German validation study of the HEAS [10], however, no sociodemographic differences in eco-anxiety were analyzed. For this reason, one aim of this study is to build on previous research findings and close the existing gap in German-speaking countries.

### Eco-anxiety and climate change anxiety

The inconsistent conceptualization of the construct anxiety in the context of ecological crises such as climate change mentioned in the beginning also results in its different operationalization. Nevertheless, there is evidence that the dimensions of climate anxiety and eco-anxiety are closely linked [17]. Previous results show significant medium to large correlations between the Cognitive-emotional and Functional Impairment subscales of the Climate Anxiety Scale (CAS) [3] and the HEAS subscales Affective Symptoms, Rumination, Behavioral Symptoms and Personal Impact Anxiety [11, 17]. According to these results and regarding convergent validity, the HEAS is expected to have medium to high correlations with climate change anxiety measured by the CAS since both instruments measure very similar constructs.

### Eco-anxiety and psychological distress

As confirmed by a recent meta-analysis [22], there is a close negative correlation between climate anxiety and mental wellbeing. It is possible that people with existing mental disorders are more susceptible to higher levels of eco-anxiety, as the stress of eco-anxiety could exacerbate pre-existing psychological distress. In this sense, a significant relationship was found between climate change anxiety and psychological distress [23]. Furthermore, climate anxiety has been associated with increased levels of depression and generalized anxiety [3, 6] as well as insomnia [24]. According to these results, we expect general psychological distress to be associated with higher eco-anxiety. In particular, a medium to large correlation with general psychological distress as measured by the Symptom-Checklist-90^®^-Standard (SCL-90^®^-S) [25] is expected.

### Eco-anxiety and self-efficacy

Self-efficacy refers to the conviction that one’s own actions can contribute to producing effects and achieving certain goals [26]. In this sense, study results show that self-efficacy can motivate pro-environmental behavior [27], just as climate anxiety and eco-anxiety can [5, 28, 29]. However, climate change anxiety has a twofold impact on pro-environmental behaviors: a direct positive effect by acting as a motivator, or an indirect impact mediated through a negative relationship with self-efficacy [28]. As anxiety is often characterized by feelings of uncontrollability [30], self-efficacy as a general belief of controllability could be associated with lower climate and eco-anxiety. In this sense, Qin and colleagues [31] found that green self-efficacy moderates the relationship between climate change anxiety and pro-environmental behavior among Chinese adolescents. People with higher self-efficacy are more likely to believe that they can control the current situation and behave more positively when confronted with problems. On the other hand, people with low self-efficacy often have self-doubt and the feeling that they cannot cope with the current situation, which leads to psychological problems such as anxiety and worry [32]. As self-efficacy can also buffer psychological distress [33], which in turn is significantly associated with climate anxiety [23], in this study it is expected that self-efficacy relates to weaker eco-anxiety.

### Eco-anxiety and social support

Social support is associated with a wide range of (mental) health benefits. Social, supportive bonds are beneficial by helping individuals control their emotional responses to stressful situations, such as anxiety and depression, and by keeping responses to stress at low levels or by promoting faster recovery following stress [34]. In view of people’s increased anxiety during the coronavirus pandemic as another example of a global threat, Özmete and Pak [35] were able to show that anxiety levels fell significantly when perceived social support increased. After a flood disaster, a high level of perceived social support was associated with proactive coping [36]. Furthermore, results of qualitative studies suggest that social support can help reduce climate anxiety [37, 38]. Following these results, perceived social support could be seen as an aspect that can facilitate adaptation to ecological crises and reduce eco-anxiety. In this sense, in this study it is expected that social support relates to weaker eco-anxiety.

### Present study

The present study aims to evaluate the psychometric properties of the German version of the HEAS on a sample of 322 German speaking adults. Since the present sample is more heterogeneous in terms of sociodemographic characteristics than the sample of students and university staff from the German validation study by Heinzel and colleagues [10], we would like to offer a supplement to the previous results with our study. Specifically, we want to analyze sociodemographic differences in relation to eco-anxiety, as this investigation was not carried out in the German reference study [10], which could be because no sociodemographic variables other than gender and age were collected. In addition, and similar to Larionow and colleagues [12], we want to supplement the results of Heinzel and colleagues [10] by testing whether a model with a second-order factor is also suitable for the German version of the HEAS in addition to the previously proven four-factorial solution. Another novel contribution of the present study lies in investigating the predictive power of general psychological distress, self-efficacy and social support on eco-anxiety. Beyond the derived hypotheses, it is investigated which of these variables are most predictive of the experience of eco-anxiety.

## Methods

### Participants and procedure

The sample was recruited through an online survey on SosciSurvey.de. Flyers calling for participation in the study were distributed and the link to the study was disseminated via the Magdeburg-Stendal University of Applied Sciences email distribution lists and via private social media channels such as Instagram using the snowball method. The data were collected between April and July 2024. The study was accessed 466 times. Three people did not consent, 22 had more than 5% missing values and 119 did not complete the study. The data of 322 people (97 (30.1%) male, 217 (67.4%) female, 8 (2.5%) identifying as another gender^1^) whose ages ranged from 18 to 79 years (M = 36.64; SD = 14.77) could be evaluated. The complete sociodemographic characteristics of the sample are shown in Table 2.

### Measurements

**Eco-anxiety** was measured using the German version of the Hogg Eco-Anxiety Scale (HEAS) [10], originally developed by Hogg and colleagues [7]. The HEAS is an instrument comprising 13 items to measure the level of eco-anxiety that participants have experienced in the last two weeks using a 4-point frequency rating scale (0 = *not at all*, 1 = *several of the days*, 2 = *over half the days*, 3 = *nearly every day*). The HEAS consists of the four dimensions: affective symptoms (α = 0.87, ω = 0.86), rumination (α = 0.84, ω = 0.84), behavioral symptoms (α = 0.79, ω = 0.79), and anxiety about one’s personal impact on the planet (α = 0.90, ω = 0.90).

**Climate Anxiety** was measured with the German adaptation of the Climate Anxiety Scale (CAS) [6] with a seven-point Likert scale from 1 = *does not apply at all* to 7 = *applies completely*. The 13 item-scale originally developed by Clayton and Karazsia [3] is divided into the subscales *cognitive-emotional impairment* and *functional impairment*. According to the authors, these two factors form the core of climate anxiety. In line with their interpretation, in this study the other two factors *experience of climate change* and *behavioral engagement*, which were also assessed in their original study, are regarded rather as potential correlates of climate anxiety. In their validation study, Wullenkord and colleagues [6] were unable to replicate the original two-factorial structure of the CAS and used a single score. Therefore, in this study a total score of climate anxiety by averaging all 13 items of the CAS is calculated and used for all analyses (α = 0.90, ω = 0.90).

**Table 2:** Demographic characteristics of the sample

Variable	*n*	%
Sex		
Male	97	30.1
Female Divers	217 8	67.4 2.5
Age groups, years		
18–24	85	26.4
25–34	91	28.3
35–44	52	16.1
45–54	38	11.8
55–64	45	14.0
≥65	11	3.4
Marital status		
Single^a^	218	67.7
Married	104	32.3
Parental status		
Parents	117	36.3
Not parents	205	63.7
Education		
A-Levels and below	133	41.3
Completed degree	189	58.7
Employment		
Yes^b^	181	56.2
No	141	43.8
Net household income		
< 2.000€	140	43.5
≥ 2.000€	182	56.5
Nationality		
German	312	96.9
Other	10	3.1
Country		
Germany	309	96.0
Austria	4	1.2
Switzerland	9	2.8
Federal state		
East	72	23.3
West	237	76.7
Confession		
Yes	114	35.4
No	208	64.6
Student		
Yes	124	38.5
No	198	61.5

^a^(includes divorced and widowed); ^b^(includes part-time employed)

**Psychological distress** was assessed by the SCL-90^®^-S [25]. The instrument maps psychological distress that participants have experienced in the last seven days on the following scales: Somatization (SOM), Obsessive-Compulsive (O-C), Interpersonal Sensibility (I-S), Depression (DEP), Anxiety (ANX), Hostility (HOS), Phobic Anxiety (PHOB), Paranoid Ideation (PAR) and Psychoticism (PSY). Participants are asked to indicate on a five-point Likert scale (0 = *not at all* to 4 = *extremely*) the extent to which they have been distressed by the stated complaints. The Global Severity Index (GSI) as an indicator of general psychological distress showed excellent internal consistency (α = 0.97, ω = 0.97).

**Social support** was measured using the German version of the Oslo Social Support Scale (OSSS-3) [39], which consists of three items assessing the level of social support. The internal consistency (α = 0.59, ω = 0.61) could be regarded as acceptable.

**General self-efficacy** was measured with the General Self-Efficacy Short Scale (ASKU) [40] which is a self-assessment tool for recording subjective competence expectations. Participants answer the three items on a five-point rating scale (1 = *doesn’t apply at all* to 5 = *applies completely*). The reliability coefficients showed good internal consistency (α = 0.87, ω = 0.87).

### Data analysis

Statistical analyses were conducted using IBM SPSS Statistics 29 and AMOS 29. The descriptive statistical analysis included calculating the mean, standard deviation, skewness, and kurtosis for the HEAS items and scales. Student’s and Welch’s t-test for independent samples were used to assess differences between groups across different sociodemographic variables in the HEAS and its subscales. Pearson’s correlation and point biserial correlation coefficient was used to assess correlations between variables. A multiple regression analysis was conducted to explore the influence of psychological distress, self-efficacy and social support on eco-anxiety. The stepwise procedure was chosen, in which the variables are sequentially included in the model in a similar way to forward selection [41]. The independent variable that correlates most strongly with the dependent variable is added to the model first and additionally tested at each step to determine whether the least “useful” variable should be removed. To estimate internal consistency, Cronbach’s Alpha and McDonald’s Omega were calculated for the HEAS and its subscales. To replicate the frequently documented four-factorial structure of the HEAS, a confirmatory factor analysis (CFA) was performed. Our sample size was sufficient for Confirmatory Factor Analysis (CFA; *N* > 200 [42]). Also, a second-order factor analysis was conducted to check for the presence of a second-order factor called “eco-anxiety”. The model fit was evaluated following the recommendations of Schermelleh-Engel and colleagues [43]: good (acceptable) model fit is given with Santorra-Bentler χ^2^/df index below 2.0 (below 3.0), Goodness-of-Fit Index (GFI) above 0.95 (above 0.90), Comparative Fit Index (CFI) as well as Tucker-Lewis-Index (TLI) above 0.97 (above 0.95), Standardized Root Mean Square Residual (SRMR) below 0.05 (below 0.10), and Root Mean Square Error of Approximation (RMSEA) below 0.05 (below 0.08). The models’ validity was further examined using the following statistics: composite reliability (CR), average variance extracted (AVE), and heterotrait-monotrait ratio (HTMT) [44–46]. According to Hair Jr, Howard and Nitzl [47], CR values should range between 0.70 and 0.95, while AVE values should be higher than 0.50. To achieve satisfactory discriminant validity, all HTMT ratios should be lower than 0.85 [48].

## Results

### Factor structure of the German HEAS

**Fig. 1:**
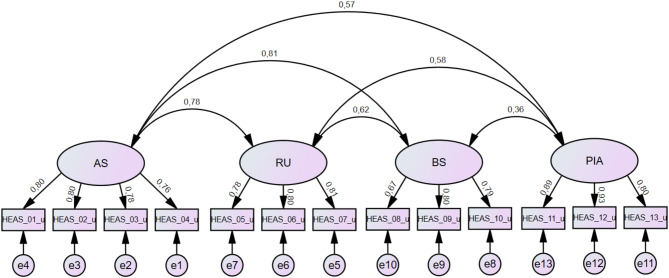
Graphical structure of the four-factorial model

Testing the four-factorial model in a confirmatory factor analysis showed that the model fit the data acceptably (χ^2^ (59) = 167.901, *p* <.001). Only the fit index SRMR indicates a good model fit, while the fit indices χ^2^/df, GFI, CFI and RMSEA reflect an acceptable model fit and the TLI is below an acceptable value [42] (Table 3). As seen in Fig. 1, the factor loadings on the respective factors were medium to high for all items (0.67-0.93). As seen in Table 4, the CR values range between 0.82 and 0.91 [47], all AVE values are higher than 0.50 [47], and all HTMT ratios are lower than 0.85 [48], so that the requirements are met.

**Fig. 2:**
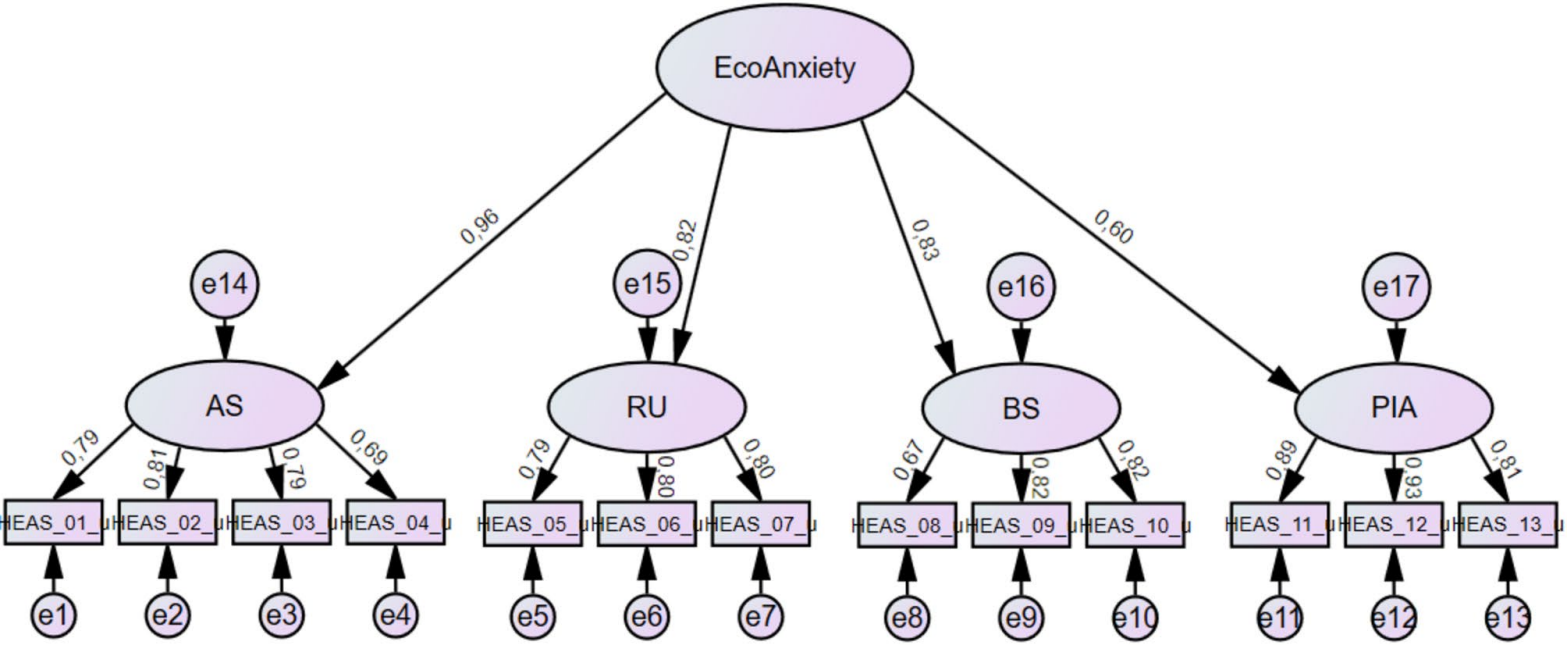
Graphical structure of the second order model

The second-order model (Fig. 2) was identified but did not have satisfactory fit (χ^2^ (64) = 209.21, *p* <.001). While the GFI, RMSEA and SRMR were acceptable, the other fit indices were rather low and indicated poorer model fit compared to the four-factorial model [42] (Table 3).

**Table 3:** Goodness-of-fit indices for the four-factor model and second-order model

	χ^2^-Test	χ^2^/df	GFI	CFI	TLI	RMSEA (90% CI)	SRMR
four-factor-model	χ^2^ = 167.90 df = 59 *p* <.001	2.85	0.93	0.96	0.94	0.08 (0.06-0.09)	0.04
second-order-model	χ^2^ = 209.21 df = 64 *p* <.001	3.27	0.91	0.94	0.93	0.08 (0.07-0.10)	0.06

GFI: Goodness-of-Fit Index. CFI: Comparative Fit Index. TLI: Tucker-Lewis Index. RMSEA: Root mean standard error of approximation. CI: Confidence Interval. SRMR: Standardised root mean squared residual

**Table 4:** Structural validity analysis of the four-factorial model

Factor	CR	AVE	HTMT Ratio		
			AS	RU	BS
AS	0.86	0.62			
RU	0.84	0.64	0.78		
BS	0.82	0.57	0.81	0.63	
PIA	0.91	0.77	0.57	0.58	0.36

AS: Affective Symptoms, RU: Rumination, BS: Behavioral Symptoms, PIA: Personal Impact Anxiety, CR: Composite Reliability, AVE: Average Variance Extracted, HTMT: Heterotrait-Monotrait

### Reliability analysis

Every HEAS-subscale showed good internal consistency with Cronbach’s α = 0.87 and McDonald’s ω = 0.86 for Affective Symptoms, α = 0.84 and ω = 0.84 for Rumination, α = 0.79 and ω = 0.79 for Behavioral Symptoms and α = 0.90 and ω = 0.90 for Personal Impact Anxiety. The HEAS total scale showed excellent internal consistency with α = 0.91 and ω = 0.91.

### Descriptive data

Table 5 shows the psychometric characteristics of all 13 items of the HEAS, the total scale and subscales. As seen in Table 5, the reliability of the individual scales would not improve if one of the respective items were removed. The mean values are all between 0 (= not at all) and 1 (= several of the days) and are therefore in the lower range of the scale from 0 to 3. Except for items 4, 11, 12, 13 and the Rumination subscale, which are normally distributed in terms of skewness because they lie in the range from − 1 to + 1, all other skewness values are above + 1, which indicates a right-skewed distribution of the data for the respective items and scales. The kurtosis values for items 5, 8, 9, 10 and the Behavioral Symptoms subscale are above + 3, which indicates a peaked distribution compared to the normal distribution curve. For all other items and scales, the kurtosis values can be regarded as normally distributed, as they lie in the range from − 3 to + 3. Based on the item discrimination values, it can be assumed that all the items measure something similar to the HEAS in general. The item difficulty values indicate a low tendency to agree to the item statements, which can be linked to the right-skewed distribution of the data.

**Table 5:** Psychometric characteristics of the HEAS items and scales

Item	M	SD	S	K	r_rit_	p_i_	α if item removed	ω if item removed
HEAS total scale (α =.91, ω =.91)								
	0.55	0.50	1.19	1.39				
Subscale Affective Symptoms (α =.87, ω =.86)								
	0.53	0.59	1.56	2.94				
1	0.62	0.74	1.25	1.70	.72	20.70	.82	.83
2	0.41	0.67	1.74	3.08	.71	13.66	.83	.83
3	0.49	0.71	1.53	2.25	.69	16.25	.83	.84
4	0.61	0.70	1.02	1	.68	20.50	.83	.83
Subscale Rumination (α =.84, ω =.84)								
	0.39	0.55	1.57	2.44				
5	0.38	0.64	1.84	3.48	.66	12.53	.80	
6	0.33	0.57	1.75	3.07	.62	10.97	.74	
7	0.48	0.68	1.47	2.15	.67	15.84	.78	
Subscale Behavioral Symptoms (α =.79, ω =.79)								
	0.33	0.54	2.20	5.30				
8	0.39	0.72	1.96	3.49	.49	13.15	.78	
9	0.34	0.61	1.87	3.35	.65	11.28	.72	
10	0.26	0.60	2.70	7.68	.55	8.59	.65	
Subscale Personal Impact Anxiety (α =.90, ω =.90)								
	0.94	0.80	0.68	-0.10				
11	0.93	0.86	0.67	-0.21	.64	30.85	.86	
12	0.94	0.86	0.72	-0.02	.63	31.26	.82	
13	0.95	0.90	0.72	-0.21	.59	31.78	.90	

Note: Omega if item removed cannot be estimated for less than 4 items. M: mean. SD: standard deviation. S: skewness. K: kurtosis. r_it_: Item Discrimination. p_i_: Item Difficulty. α: Cronbach’s Alpha. ω: McDonald’s Omega

### Correlations of German HEAS

Correlational analyses showed that several variables were related to eco-anxiety (see Table 6). All HEAS subscales as well as the HEAS total scale correlated significantly high with the CAS. Furthermore, all HEAS subscales and the total scale correlated significantly medium to high with the GSI of the SCL-90^®^-S, thus indicating convergent validity. Regarding divergent validity, significant low negative correlations were found between the OSSS-3 and the Rumination and Behavioral Symptoms subscales and the HEAS total scale, while the Affective Symptoms and Personal Impact Anxiety subscales did not correlate significantly with the OSSS-3. Regarding general self-efficacy, significant low negative correlations were found between the ASKU and all scales, except Personal Impact Anxiety with no significant correlation. The HEAS total scale was unrelated to sex (0 = male, 1 = female), age, marital status (0 = single/divorced/widowed, 1 = married), education (0 = A-Levels and below, 1 = completed degree), confession (0 = yes, 1 = no), and student status (0 = student, 1 = no student), whereas there were significant small correlations with the variables parental status (0 = parents, 1 = not parents), employment (0 = yes, 1 = no), net household income (0 = < 2.000 €, 1 = ≥ 2.000 €) and federal state (0 = East, 1 = West), indicating that eco-anxiety relates to having no children, being unemployed, having a net household income of ≥ 2.000 € and living in Western Germany. Regarding the subscales, having affective symptoms of eco-anxiety relates to having no children, having a completed degree, being unemployed, having a net household income of ≥ 2.000 € and living in Western Germany; having behavioral symptoms relates to having a completed degree, having a net household income of ≥ 2.000 € and living in Western Germany; feeling anxious about the own negative impact on the planet relates to younger age, being female, being single/divorced/widowed, having no children and living in Western Germany. There were no significant correlations between the subscale Rumination and the sociodemographic variables.

**Table 6:** Bivariate correlations between variables (Pearson and point biserial)

	Affective symptoms	Rumination	Behavioral symptoms	Personal impact anxiety	HEAS total scale
Affective symptoms					
Rumination	0.67***				
Behavioral symptoms	0.68***	0.50***			
Personal impact anxiety	0.51***	0.51***	0.31***		
HEAS total scale	0.89***	0.81***	0.74***	0.76***	
CAS	0.64***	0.61***	0.52***	0.56***	0.73***
CAS cognitive-emotional impairment	0.58***	0.53***	0.46***	0.49***	0.64***
CAS functional impairment	0.61***	0.60***	0.50***	0.56***	0.71***
SCL-90^®^-S Global Severity Index	0.52***	0.37***	0.47***	0.34***	0.53***
OSSS-3	− 0.11	− 0.12*	− 0.14*	− 0.03	− 0.12*
ASKU	− 0.20***	− 0.13*	− 0.21***	− 0.09	− 0.19***
Age	− 0.11	− 0.001	− 0.01	− 0.11*	− 0.08
Sex	0.10	0.02	0.05	0.13*	0.10
Marital status	− 0.07	0.04	− 0.02	− 0.12*	− 0.06
Parental status	0.15**	0.06	0.10	0.16**	0.15**
Education	0.13*	0.02	0.12*	0.001	0.08
Employment	0.17**	0.06	0.06	0.10	0.13*
Net household income	0.20***	0.05	0.11*	0.10	0.15**
Federal state	− 0.13*	− 0.11	− 0.13*	− 0.12*	− 0.15**
Confession	− 0.08	− 0.06	− 0.07	0.04	− 0.05
Student status	− 0.11	0.03	− 0.07	− 0.06	− 0.07

* *p* <.05, ** *p* <.01, *** *p* <.001

### Sociodemographic and psychological influences on eco-anxiety

Differences in the HEAS dimensions between men and women, between people of 32 years of age or younger and people older than 32– according to median split–, and between participants with and without children were explored. It was found that women reported a significantly higher mean value than men on the scale Personal Impact Anxiety (t_(312)_ = 2.35, *p* =.02, d = 0.29). No significant differences between the two different age groups across all HEAS scales were found. People who did not (yet) have children at the time of the survey gave higher means on the HEAS total scale (t_(320)_ = 2.73, *p* =.007, d = 0.32) and the subscales Affective Symptoms (t_(320)_ = 2.64, *p* =.009, d = 0.31) and Personal Impact Anxiety (t_(320)_ = 2.87, *p* =.004, d = 0.33) than people who were already parents.

To gain a better understanding of the relationships, a stepwise multiple linear regression was conducted to understand how the variables of interest– general self-efficacy, general psychological distress, and social support– predict eco-anxiety. We found no evidence for multicollinearity and singularity (VIF < 10). The final model was reached after one step by including the GSI of the SCL-90^®^-S (see Table 7). The multiple linear regression model indicates that of these three variables only the GSI has a statistically significant influence on the eco-anxiety criterion (F_(1,320)_ = 123.227, *p* <.001, *N* = 322). The coefficient of determination (R^2^) was 0.278 (R^2^_adjusted_ = 0.276), which represents a small correlation between predicted and actual values [49]. In this sense, about 28% of the variance in eco-anxiety could be explained by psychological distress (β = 0.527, t_(320)_ = 11.101, *p* <.001), which indicates a strong variance explanation [49].

**Table 7:** Multiple regression model

	Estimate (B)	SE	β	t	*p*	Tolerance	VIF
Intercept	0.223	0.038		5.916	< 0.001		
SCL-90^®^-S GSI	0.561	0.051	0.527	11.101	< 0.001	1.000	1.000
ASKU	-	-	-0.02	-0.394	0.694	0.892	1.121
OSSS-3	-	-	0.031	0.624	0.533	0.922	1.084

SE: standard error. VIF: Variance inflation factor

## Discussion

This study was concerned with the psychometric testing of an existing German version of the HEAS [10] on a sample of 322 German-speaking adults. In line with the validation study of the original instrument [7] as well as with the German version [10] and other validated adaptations [8–18], this study’s CFA found evidence that the HEAS consists of the four dimensions Affective Symptoms, Rumination, Behavioral Symptoms, and Personal Impact Anxiety. Checking for the existence of a second-order factor called “eco-anxiety” with the four dimensions as first order factors for the German HEAS resulted in an inferior model fit compared to the four-factor solution, which is in line with the previous finding of Larionow and colleagues [12] and confirms the multidimensionality of the construct, so that even in the German version the construct is better represented by its four facets than by a general factor, which nevertheless has a very good reliability. Regarding the answers to the HEAS items, the present sample showed a low tendency to agree with the individual statements, which is also reflected in the low mean values and the right-skewed distribution of the data. This result could be interpreted to mean that eco-anxiety is not very widespread in this sample.

According to the Point biserial correlation analysis, it was noticeable that the sociodemographic variables correlated differently with the HEAS total scale and its dimensions. However, it should be noted that the correlations were only slightly significant in each case and there were no significant correlations with the Rumination subscale. Age, sex and marital status were only correlated with the Personal Impact Anxiety subscale, suggesting that younger single (or divorced/widowed) females are more likely to feel anxious about their personal negative impact on the planet. Not having children was associated with more affective symptoms, more anxiety about the own negative impact on the planet and overall, more eco-anxiety. Having a degree was associated with more affective and behavioral symptoms, being unemployed was associated with more affective symptoms and eco-anxiety in general, having a net household income ≥ 2.000 € was associated with more affective and behavioral symptoms as well as eco-anxiety in general, and living in Western Germany was associated with eco-anxiety and its dimensions except for Rumination. The results of the analysis of sociodemographic differences suggest that eco-anxiety is higher amongst certain groups. The finding that women report significantly more anxiety about their personal negative impact on the planet than men is in line with previous results [11, 14, 16, 18]. However, in the present study no significant gender differences on the Affective Symptoms and Rumination subscales found in other studies [14, 16, 18] occured. In contrast to previous results, which reported significantly higher scores for younger people on the scales Affective Symptoms, Personal Impact Anxiety [11, 14] and Behavioral Symptoms [14], just a significant small negative correlation between age and the subscale Personal Impact Anxiety was found in the present study, suggesting that younger participants were more afraid of their own negative impact on the planet. Moreover, no significant differences on the HEAS subscales between people under and over 32 years of age were found. This could be compared with Türkarslan and colleagues [18], who also found no significant differences between people under and over 25 years of age, but the different age limit of the groups must be considered. The greater reported anxiety about their own negative influence on the planet among participants who did not (yet) have children at the time of the survey compared to parents may indicate that these people are reconsidering their reproductive intentions. Even though no significant differences were found regarding the HEAS total scale, this can be partially compared with the result of Jalin and colleagues [20] who found that not having children predisposed to a higher level of eco-anxiety.

As expected, all HEAS subscales and the total scale were strongly positively associated with the CAS, illustrating their conceptual relation. Also, the dimensions of the German HEAS showed significant positive large correlations with the GSI of the SCL-90^®^-S, suggesting the link between eco-anxiety and psychological burden. Treating these associations as bi-directional, people more predisposed to general psychological distress could manifest this in many domains, like ecological aspects. On the other hand, people’s eco-anxiety could contribute to general psychological distress. Between the German HEAS dimensions and the OSSS-3 significant negative small correlations were only found for the subscales Rumination and Behavioral Symptoms, suggesting that ruminating more and showing more behavioral symptoms is associated with less social support. The results of the multiple linear regression analysis showed that social support was no significant predictor of eco-anxiety. Following results that climate anxiety is associated with lower self-efficacy [50], also in this study significant small negative correlations between the HEAS subscales (except Personal Impact Anxiety) and the ASKU were found. Analogous to climate anxiety, eco-anxiety may lead to negative thoughts about global warming and feelings of hopelessness and helplessness, thus negatively impacting general self-efficacy [28]. The results of the multiple linear regression analysis showed that self-efficacy was no significant predictor of eco-anxiety, indicating that the idea of increasing self-efficacy as an intervention to alleviate eco-anxiety could not be supported. In this sense, Schönfeld and colleagues [51] also claim that the role of self-efficacy is not uniformly beneficial and that higher levels of self-efficacy can sometimes lead to an increase in neuroendocrine and psychological stress responses which in turn means that therapeutic interventions to alleviate psychological stress may not always promote self-efficacy in principle and that high levels of self-efficacy may even be detrimental or harmful.

### Implications and limitations

The practical implications of this study are important for both researchers and practitioners. By ensuring the reliability and validity of the instrument, accurate measurement of the construct is facilitated. This has practical implications for practitioners who will increasingly work with people affected by eco-anxiety in the context of advancing climate change, as they will have a validated measurement tool with which to assess the extent of this type of response to ecological crises. Nevertheless, when interpreting this study’s results, some limitations need to be considered. First, this study’s sample was not representative for the general population given that most participants were young, female, and highly educated, because of the predominant recruitment in the university setting. To assess whether our results can be generalized to the German speaking population, future research should be based on more representative samples. Furthermore, when interpreting the results of the correlational analysis and independent samples t-tests, however, the uneven distribution of the sample into the dichotomous groups of sociodemographic variables must be considered. Also, as it was a cross-sectional study, determinations of the cause-and-effect relationship between eco-anxiety and its correlates are limited. Since we have only mapped the reliability via the internal consistency, an extension of our results could be to test the stability of the construct via test-retest reliability.

## Conclusions

The German version of the HEAS, originally from Heinzel and colleagues [10], was tested in a sample of 322 German speaking participants. The HEAS total scale and subscales were found to be internally consistent. Performing a CFA, it was possible to confirm the four-factor structure, while the second-order model showed an inferior fit. Following this, the multidimensionality of the structure is supported, but for practical reasons, nevertheless, a total score for the construct of eco-anxiety could be considered, which shows an excellent internal consistency and is therefore a reliable indicator for measuring eco-anxiety in general. The result of the multiple linear regression analysis showed that when considering the variables psychological distress, self-efficacy, and social support, only psychological distress emerges as a significant predictor of eco-anxiety.

## Data Availability

The datasets used and/or analysed during the current study are available from the corresponding author upon reasonable request.

## Footnotes

1 The number of people who identified as a gender other than male or female was too small to allow statistical analysis, so their data were not included in analyses that took gender into account (i.e., correlations).

## Abbreviations

ANX: Anxiety
AS: Affective Symptoms
ASKU: General Self Efficacy Short Scale
AVE: Average Variance Extracted
BS: Behavioral Symptoms
CAS: Climate Anxiety Scale
CFA: Confirmatory Factor Analysis
CFI: Comparative Fit Index
CI: Confidence Interval
CR: Composite Reliability
DEP: Depression
GSI: Global Severity Index
HEAS: Hogg Eco-Anxiety Scale
HOS: Hostility
HTMT: Heterotrait-Monotrait
I-S: Interpersonal Sensibility
K: Kurtosis
M: Mean
O-C: Obsessive-Compulsive
OSSS-3: Oslo Social Support Scale
p_i_: Item Difficulty
PAR: Paranoid Ideation
PHOB: Phobic Anxiety
PIA: Personal Impact Anxiety
PSY: Psychoticism
r_it_: Item Discrimination
RMSEA: Root mean standard error of approximation
RU: Rumination
S: Skewness
SCL-90®-S: Symptom-Checklist-90^®^-Standard
SD: Standard deviation
SE: Standard error
SOM: Somatization
SRMR: Standardised root mean squared residual
TLI: Tucker-Lewis Index
VIF: Variance inflation factor
